# Multidisciplinary Management of Patients with Unresectable Hepatocellular Carcinoma: A Critical Appraisal of Current Evidence

**DOI:** 10.3390/cancers11060873

**Published:** 2019-06-22

**Authors:** Pierre M. Gholam, Renuka Iyer, Matthew S. Johnson

**Affiliations:** 1 Liver Center of Excellence, University Hospitals Cleveland Medical Center, 11100 Euclid Avenue WRN5066, Cleveland, OH 44106, USA; 2 Department of Medicine, Roswell Park Cancer Institute, Buffalo, NY 14203, USA; Renuka.Iyer@RoswellPark.org; 3 Department of Radiology, Indiana University School of Medicine, Indianapolis, IN 46202, USA; matjohns@iupui.edu

**Keywords:** hepatocellular carcinoma, locoregional treatment, TACE, TARE, systemic treatment

## Abstract

Hepatocellular carcinoma (HCC) is a leading cause of new cancer diagnoses in the United States, with an incidence that is expected to rise. The etiology of HCC is varied and can lead to differences between patients in terms of presentation and natural history. Subsequently, physicians treating these patients need to consider a variety of disease and patient characteristics when they select from the many different treatment options that are available for these patients. At the same time, the treatment landscape for patients with HCC, particularly those with unresectable HCC, has been rapidly evolving as new, evidence-based options become available. The treatment plan for patients with HCC can include surgery, transplant, ablation, transarterial chemoembolization, transarterial radioembolization, radiation therapy, and/or systemic therapies. Implementing these different modalities, where the optimal sequence and/or combination has not been defined, requires coordination between physicians with different specialties, including interventional radiologists, hepatologists, and surgical and medical oncologists. As such, the implementation of a multidisciplinary team is necessary to develop a comprehensive care plan for patients, especially those with unresectable HCC.

## 1. Introduction

Cancers of the liver and bile duct, of which hepatocellular carcinoma (HCC) is the most common type, are expected to be the 13th leading cause of new cancer diagnoses in men in the United States in 2019 [1]. An estimated 31,780 deaths due to these cancers are anticipated, making them the fifth leading cause of cancer-related mortality in men and seventh leading cause in women [2]. Unlike many other common cancer types, incidence of HCC is expected to continue to rise, with a similar increase in the rate of deaths due to HCC.

The etiology of HCC is varied, with a range of different presentations and natural histories [3]. Approximately 85% of patients diagnosed with HCC have cirrhosis of the liver, although patients infected with the hepatitis B virus (HBV) or with aflatoxin B1 exposure may develop HCC without underlying cirrhosis, which is rarely the case for patients infected with the hepatitis C virus (HCV). Non-alcoholic steatohepatitis, also known as fatty liver disease, is another common cause of cirrhosis that can progress to HCC. Development of hepatic malignancy has also been linked to alcohol use and the presence of certain genetic mutations.

At the level of the individual patient, concomitant cirrhosis and the number, size, and location of hepatocellular tumors will affect the treatment approach. In addition, multiple disease-related factors need to be taken into account, such as the presence of vascular involvement or extrahepatic disease, when deciding on the best treatment options for these patients. Consequently, a multidisciplinary approach involving several physicians with different specialties (e.g., diagnostic and interventional radiologists, surgical oncologists, hepatologists, and medical oncologists) is necessary to determine the best approach to treatment and maximize potential outcomes for patients with HCC.

## 2. The Evolving Treatment Paradigm for Patients with Unresectable HCC

Surgery or liver transplant are preferred options for patients with HCC as they can be curative, but approximately 70% of patients are diagnosed at a stage that prohibits these treatments [4,5,6,7,8]. Transplant may become an option for down-staging therapy as a bridge to permit some patients who could not initially undergo transplant to eventually become transplant candidates if they have durable responses with a reduction in number or size of tumors to within acceptable criteria. Multiple guidelines have been created to address the treatment of patients with HCC who cannot undergo resection or transplant (Table 1). Many different treatment modalities have been endorsed in these guidelines, with treatment selection based upon both patient and disease characteristics. Consequently, a multidisciplinary approach is necessary to ensure that the guidelines are adhered to and patients are treated with the right modalities at the right time.

### 2.1. Locoregional Therapy

Locoregional treatments are a set of therapeutic approaches that directly target tumors in the liver. Among the locoregional modalities, ablative therapies are commonly used for tumors that can be accessed without damaging surrounding structures [6]. Outcomes for patients with tumors <2 cm who undergo ablation are generally similar to those having surgery [4]. 

Transarterial chemoembolization (TACE) is another locoregional modality that involves the local delivery of chemotherapy to the tumor and is generally recommended for patients with liver-limited disease [4,5,6,7,8]. Several randomized trials have been conducted to examine the efficacy and safety of TACE. In one notable study, patients with untreated hepatocellular tumors who were not suitable for curative treatments and who had Child–Pugh A or B liver function were randomized to arterial embolization, TACE, or symptomatic treatment (N = 112) [9]. Results showed that the probability of survival at 1, 2, and 3 years was highest for the TACE group. In addition, median overall survival (OS) was significantly longer in the TACE group relative to the symptomatic treatment group (25.3 months vs. 17.9 months, *p* = 0.009; comparison to the embolization group was not made because the trial was stopped early). A similar study was conducted in 79 Asian patients with newly diagnosed HCC where the estimated 1-, 2-, and 3-year survival rates were 57%, 31%, and 26%, respectively, in the TACE group, compared with 32%, 11%, and 3%, respectively, for the symptomatic treatment control group [10]. The relative risk of death was significantly lower in the TACE group (RR 0.50 [95% CI 0.31–0.81; *p* = 0.005). 

These results showed important survival benefits of the procedure, but it is important to keep in mind that prognosis after TACE in virtually all patients is eventually limited by progression of liver disease or cancer. Consequently, TACE failure has been defined in several different ways, including lack of substantial necrosis by mRECIST criteria after two rounds, failure of follow-up treatment to induce necrosis in progressing sites, major progression (defined as substantial liver involvement, vascular invasion, and/or extrahepatic spread) after an initial response, deterioration to Child–Pugh C liver function, or poor tolerance. Unfortunately, no uniform consensus exists on the definition of “TACE failure” [4,6]. 

Transarterial radioembolization (TARE) is another locoregional therapy option for patients with liver-dominant disease in which microspheres laden with yttrium-90 (Y-90) are used to deliver radiation. Two types of Y-90–laden microspheres are available in the United States: resin spheres (SIR-Spheres^®^; Sirtex Medical Limited; North Sydney, Australia) in which the microspheres are coated with Y-90, and glass spheres (TheraSphere^®^; BTG International Medicine; London, UK) in which the isotope is an intrinsic component of the microsphere. Recent data showed no improvements in survival but superior local tumor control through TARE with Y-90 resin microspheres relative to systemic therapy with sorafenib (NEXAVAR^®^; Bayer, Whippany, NJ, USA) in heavily pretreated patients with liver-only disease [11,12]. Although TARE was better tolerated, hazard ratios for OS (which were similar in both trials and trended toward better survival with sorafenib) showed no evidence of superiority to sorafenib in terms of OS; however, the design of the trial may have contributed to this finding. Specifically, the inclusion of patients who had already progressed on ≥2 rounds of TACE essentially selected for patients who had already failed locoregional therapy. Prior TACE may have also increased the likelihood that patients had vascular occlusion, which would have limited the effectiveness of subsequent TARE. Several patients randomized to TARE did not get the intended therapy because they lacked access to a site that could perform the procedure in Asia, which confounded the analysis of the intent-to-treat sample. 

### 2.2. Radiation Therapy

External beam radiation therapy (EBRT) may also be considered for locoregional treatment of hepatocellular tumors. Guidelines from the National Comprehensive Cancer Network (NCCN) recommend that EBRT be considered for all unresectable tumors [6]. EBRT may also be useful for symptom control in patients with metastatic disease [6], although this remains controversial. Additional data are needed to further clarify the role of EBRT in the treatment of patients with unresectable HCC.

### 2.3. Systemic Therapy

Systemic therapy has traditionally been thought of as an option for patients who are not suitable for locoregional therapies or who have extensive intra- or extrahepatic disease [4,5,6,7,8]. This largely confines this modality to BCLC STAGE C patients. As of the time of writing of this review, there are now six FDA-approved systemic therapies for unresectable HCC, up from only sorafenib merely three years ago. These include sorafenib and lenvatinib in front line as well as regorafenib, nivolumab, pembrolizumab, ramucirumab and cabozantinib in persons previously treated with sorafenib. Survival data for persons treated with multiple lines of therapy are emerging. The following is a critical appraisal of the evidence that led to these approvals and a perspective on where they fit in the current treatment arsenal of unresectable HCC. 

### 2.4. Front-Line Therapies

For the last 12 years, sorafenib has been the mainstay of systemic treatment for patients with HCC based on the results of 2 randomized phase 3 clinical trials. The SHARP trial primarily enrolled patients from Europe and North/Central/South America with advanced-stage (Barcelona Clinic Liver Cancer [BCLC] stage C) disease and Child–Pugh A liver function, and ECOG PS ≤2 who were not eligible for surgical or locoregional therapy [13]. The results showed that median OS was 10.7 months in the sorafenib-treated group, compared with 7.9 months in the placebo-treated group (HR 0.69 [95% CI 0.55 to 0.87], *p* < 0.001). 

Lenvatinib (LENVIMA^®^; Eisai, Inc.; Woodcliff Lake, NJ, USA) is a tyrosine kinase inhibitor that primarily targets VEGFR-1,2,3, as well as FGF and other pathways that have been implicated in tumor angiogenesis and proliferation, including RET, KIT, and PDGFRα [14]. The REFLECT trial was a randomized, open-label trial comparing lenvatinib with sorafenib in patients with unresectable HCC who had not previously been treated with systemic therapy. While the majority of enrolled patients had BCLC C disease, the trial excluded potential patients with tumor burden exceeding 50% of total liver volume, tumor invasion of a large bile duct, and main portal vein invasion (branch portal vein invasion was allowed). This was done to align with the Japanese Society for Hepatology guidelines for systemic therapy in unresectable HCC in the setting of a heavy recruitment in Asia (and Japan in particular). Other key enrollment criteria included Child–Pugh class A liver function, ECOG PS 0 or 1, <50% liver occupation, and the absence of portal vein invasion. The study met its primary endpoint of OS which was shown to be non-inferior to sorafenib. Median OS duration was 13.6 months (95% CI 12.1–14.9). In addition, there was a 2.5-times increase in duration of median PFS and a 3-times increase in objective response rate with lenvatinib. While AEs appeared comparable between the sorafenib and lenvatinib arms, there was more hypertension but hand foot skin reaction, which often leads to discontinuation of treatment, was less common in the lenvatinib group. Interestingly, the OS in both arms of this trial was slightly longer than that seen with other first-line trials in HCC [14]. This may have been due to the relatively high percentage of patients who received additional systemic and/or non-systemic treatment after progression.

### 2.5. Systemic Therapy after Sorafenib

The Food and Drug Administration (FDA)-approved indications for all four approved systemic therapies beyond front line state that they are approved for the treatment of patients “who have been previously treated with sorafenib”. This has largely been interpreted as “subsequent line” by treaters and experts although this issue is debatable in the absence of data on patients who have had prior treatment with lenvatinib or immunotherapy.

Regorafenib (STIVARGA^®^; Bayer; Whippany, NJ, USA) is an oral tyrosine kinase inhibitor that targets multiple protein kinases involved in tumor oncogenesis, angiogenesis, metastasis, and immunity, including RET, VEGFR1-3, KIT, PDGFRα and β, FGFR1 and 2, RAF-1, BRAF, and CSF1R [15]. Enrolled patients had previous treatment with sorafenib at a dose of ≥400 mg/d for ≥20 of the 28 days prior to detection of progressive disease (intolerability was an exclusion) as well as BCLC class B or C disease and Child–Pugh A liver function. The RESORCE trial showed superiority of regorafenib vs. placebo (median OS 10.7 vs. 7.8 months; HR 0.63 (95% CI 0.50–0.79), *p* < 0.001) in patients who progressed on sorafenib, the majority of whom had BCLC C disease and Child–Pugh A liver function [16]. Furthermore, patients in this trial who received regorafenib demonstrated that treatment with sorafenib followed by regorafenib led to a median OS since the start of sorafenib of 26.0 months [17]. As patients were pre-selected for tolerability to sorafenib, which may have allowed them to tolerate regorafenib as well. Consequently, the efficacy of regorafenib in patients who do not tolerate sorafenib remains unknown. As expected, the AE burden of regorafenib included hand foot skin reaction, fatigue, and diarrhea most prominently.

Cabozantinib (CABOMETYX^®^; Exelixis; South San Francisco, CA, USA) is a tyrosine kinase inhibitor that targets angiogenesis pathways with shared other agents but also inhibits MET and AXL, which may impact components of tumor cell motility and metastasis [18,19]. The CELESTIAL trial enrolled a broad spectrum of patients with Child–Pugh A liver function and good performance status (ECOG 0 or 1) who had previously been treated with sorafenib [20]. Eligible patients could have also received a variety of other systemic treatments prior to enrolling, including immunotherapy agents. In fact, 27% had received two or more systemic therapies before starting the study. Patients on cabozantinib had a median OS of 10.2 months compared with 8.0 months for placebo (HR 0.76 [95% CI 0.63-0.92], *p* = 0.005). Pre-planned subset analyses showed that the benefit of cabozantinib was somewhat more pronounced in patients who received only one systemic treatment (e.g., sorafenib) prior to starting study drug. Similar to RESORCE, patients were required to have progressed on sorafenib prior to study entry and therefore the efficacy in patients who discontinue sorafenib due to intolerability is not known. In addition, the lack of an approved standard of care in second line at the time the trials were conducted meant that both RESORCE and CELESTIAL were conducted with placebo control arms. Ultimately, a trial comparing regorafenib with cabozantinib would be needed to determine which is the optimal second-line treatment option. 

Preliminary efficacy of nivolumab (OPDIVO^®^; Bristol-Myers Squibb Company; Princeton, NJ, USA) was demonstrated in the phase 1/2 CHECKMATE-040 study of sorafenib-naïve patients and patients who progressed on sorafenib treatment [21]. Eligible patients had Child–Pugh scores of ≤7 and ECOG PS 0 or 1. PD-L1 expression was not required for entry into the trial. Notably, 61% of patients who progressed on sorafenib who were then treated with nivolumab demonstrated disease control, including 4% with complete responses, 18% with partial responses, and 40% with stable disease. Disease control was reached in 66% of patients with HCV infection and 55% of patients with HBV infection (regardless of sorafenib exposure). Median OS was not reached in this cohort of patients at the time of publication. With regard to safety, 5% of patients required steroid treatment for autoimmune hepatitis. The initial results were promising enough for the FDA to grant nivolumab accelerated approval for the treatment of patients with HCC who have had prior treatment with sorafenib. The larger, phase 3, CHECKMATE-459 trial will compare the efficacy of nivolumab vs. sorafenib in the front-line setting, further helping to establish immunotherapy in the treatment of patients with HCC.

Additional preliminary results have been presented for pembrolizumab (KEYTRUDA^®^; Merck and Co, Inc; Whitehouse Station, NJ, USA) in the KEYNOTE-224 trial [22]. Patients enrolled in this trial had progressed on or were intolerant to sorafenib and had BCLC C or B disease with ECOG Ps 0 or 1 and Child–Pugh A liver function. This open-label phase 2 study also furthered the promise of immuno-oncology agents in HCC in post-sorafenib patients with an objective response rate of 17% (including one complete response). Based on these results, pembrolizumab was granted preliminary approval for the treatment of post-sorafenib patients with HCC and further investigation in two phase 3 studies is ongoing.

The anti-VEGF antibody ramucirumab (CYRAMZA^®^; Eli Lilly and Company; Indianapolis, IN) may also be prescribed in a limited population of patients with HCC based on the REACH-2 trial [23]. Patients in this trial had progressed on sorafenib or after discontinuation of sorafenib treatment and had an AFP level ≥400 ng/mL. The results showed a median OS of 8.5 months for ramucirumab (vs. 7.3 months for placebo).

Additional systemic therapy approaches are currently in development. The IMBrave150 study (NCT03434379) will assess the safety and efficacy of the combination of atezolizumab + bevacizumab relative to sorafenib in first-line HCC. The HIMALAYA study (NCT03298451) will also examine an immunotherapy combination in first line, comparing durvalumab + tremelimumab with durvalumab alone or sorafenib. Another ongoing study (NCT03164440) will assess the potential benefits of PD-1 inhibition with sintilimumab in combination with an anti-VEGF antibody IBI305 relative to sorafenib in first line HCC. In addition, an early-stage study (NCT03299946) will assess the safety and feasibility of cabozantinib + nivolumab as neoadjuvant treatment for patients with HCC. Although results have not been reported yet, it is possible that combining approaches with known benefits (i.e., PD-1 and VEGF inhibition) will lead to improved outcomes.

## 3. Multidisciplinary Care in HCC

Due to the number of different modalities utilized to treat patients with HCC, physicians with different specialties need to be involved with developing the care plan. Diagnosis of HCC is often made biochemically and radiographically, and the essential role of the pathologist is often to confirm the presence of cancer using histology, especially when there is mixed histology or the possibility of an intrahepatic cholangiocarcinoma, which has the same risk factors as HCC. Diagnostic radiologists with a focus on abdominal imaging are integral for identifying and monitoring liver lesions as well as those that have metastasized to other tissues. 

Hepatologists are likely to be treating patients for cirrhosis or other liver diseases that preceded the development of HCC and to have patients referred to them by other physicians including gastroenterologists, internists, and family practitioners. Since hepatologists have an established relationship with these patients, they are ideally placed to coordinate care, including prescribing antiviral therapies for hepatitis and systemic therapies for HCC and monitoring and treating adverse events related to both systemic and locoregional treatments. In some centers, the director of care is the hepatologist, while in others it is the medical oncologist, surgical oncologist, or interventional radiologist.

Surgeons will perform resections or work with the multidisciplinary team to identify patients who may benefit from the option of liver transplant or downstaging toward the goal of transplant. Radiation oncologists may play a role in delivering EBRT or stereotactic body radiotherapy as needed. Interventional radiologists provide input regarding efficacy, safety, and feasibility of locoregional therapies to treat tumors in the liver as well as contribute to the determination of the ideal timing of sequential therapies or other options required to control disease. Systemic therapy is managed by hepatologists or medical oncologists who may also be able to offer a number of other treatment avenues to patients, including access to clinical trials, palliative care, and survivorship support. Throughout all stages of treatment, nurses, nurse navigators, and physician assistants provide critical support for patients through financial counseling and adverse event management.

Each of these different disciplines comes with its own unique perspectives on how to treat patients with this complex disease using the rapidly evolving treatment paradigm and growing list of options that have demonstrated clinical benefits but lack data on optimal sequencing. At the same time, they may have their own implicit biases with regard to which treatment options should be employed. Organizing a multidisciplinary team to coordinate the inputs from these different providers can help to maximize the benefits for patients while simultaneously countering these biases and developing consensus where there are gaps to support evidence-based practice.

Multiple studies have shown that disease outcomes were improved when patient care was coordinated by a multidisciplinary team (Table 2). Although quantifying the value of multidisciplinary care is difficult because one must compare against hypothetical scenarios and the confounding effects of improvements in treatment over time, these studies collectively showed that patients with HCC benefited from having their treatment regimens planned by a team consisting of different specialists. For example, patients tended to be diagnosed earlier, allowing a higher percentage to receive potentially curative treatments [24,25]. The multidisciplinary team also ensured that a high rate of patients received active treatment [26]. Ultimately, these benefits led to improved survival for patients with HCC across the disease spectrum [24,25,26,27].

Implementing a multidisciplinary tumor board requires a coordinated effort among the different stakeholders to ensure adequate representation from all the specialists who contribute to the care of patients with unresectable HCC. Each member of the team must approach patients without any preconceived ideas regarding treatment. Rather, decisions should be data- and consensus-driven.

Various models for multidisciplinary tumor boards exist. A weekly meeting where cases are briefly presented to review radiology, pathology, and laboratory findings; medical history; and comorbidities to discuss the agreed-upon care plan prior to being seen in the clinic is common at many cancer centers. A thorough review of all old scans and all potential treatment options allows for higher patient satisfaction and better documentation. In addition to helping to coordinate the patient’s care, this often also minimizes the number of visits the patient needs to make to the center. Another model for multidisciplinary care planning includes a virtual tumor board, where members are not all in one location and may participate via the internet. Similar discussions occur, but some biases may exist based on the dominant discussions at the site where the bulk of the discussants are present. Presentations that are concise and have a more focused format allow for objective discussion and review. Some centers may have algorithms to channel care/referrals, and multidisciplinary referrals occur only when cases fall outside the set algorithms. Measuring and sharing of outcomes, improving access to care, and removal of barriers to increase referrals to a multidisciplinary care center should be the focus of every HCC treatment program.

## 4. Conclusions: Moving into the Future of Treating Patients with Unresectable HCC

The treatment paradigm for patients with unresectable HCC is expected to change rapidly in the next several years. For over a decade, the treatment options for these patients have been limited to locoregional therapies, sorafenib, and best supportive care, which subsequently set patterns of referral. However, while new studies have provided more evidence-based choices in the ways this population can be treated, they also bring to light evidence gaps and new possibilities. Consequently, a more thoughtful approach to each patient is needed. Now more than ever, a multidisciplinary team is needed to improve the care of patients with unresectable HCC. 

Many institutions are developing pathways in an attempt to standardize the care of their patients. However, patients with unresectable HCC are an extremely heterogeneous population, and therefore, developing one pathway that applies to all patients is extremely challenging. A cross-functional approach to each patient provides the opportunity to utilize the expertise of the different disciplines involved in the care of these patients where no standardized approaches are available.

In addition, health care reform in the United States has placed a greater emphasis on documenting and managing comorbidities. As new treatments improve survival, the practice of oncology will see a rise in the number and severity of comorbidities in patients with unresectable HCC. In addition to these comorbidities, physicians managing these patients often must also manage multiple complications associated with underlying liver disease and (in some patients) previous treatments for HBV or HCV. Palliative care due to the presence of metastatic disease is also likely to be needed. Consequently, patients with unresectable HCC will need to be cared for by many different specialists. Instituting a multidisciplinary team to address all of these different needs should help to insure the highest quality of care, while at the same time controlling costs and maintaining patient satisfaction.

The rapidly changing landscape in HCC will also be impacted by new studies assessing the benefits of locoregional therapy on survival and a number of positive studies that were recently reported and new drug approvals expected in the coming year. Consequently, multidisciplinary discussions will become even more essential to implementing evidence-based practice decisions that require a reassessment of local therapies and when options based on non-inferiority become available with no data on optimal treatment options following progression on those agents, a knowledge gap that has been noted in the updated BCLC guidelines [28]. Furthermore, several tyrosine kinase inhibitors studied after progression on sorafenib that were found to be superior in prolonging survival when compared to placebo are expected to become available in the coming year but their efficacy relative to each other has not been assessed and their efficacy without prior sorafenib exposure is not known.

As more options become available and patients begin to be referred for systemic treatment earlier, defining progression will be critical. Optimal criteria to change treatment based on the type of therapy the patient is receiving (e.g., immune checkpoint inhibitors or tyrosine kinase inhibitors) will need careful consideration, as maximizing duration and tolerability of each therapy will be key to increasing survival in these patients. A cross-functional approach with a multidisciplinary team should help to define these criteria.

The management of hypertension, variceal surveillance, and hospitalizations that can occur during prolonged therapy and immunotherapy may also require oncologists to oversee care delivered by multiple providers. In addition, there have not been definitive studies done on impact of treating HCV in patients with advanced HCC; such treatments are done on a case-by-case basis and require multidisciplinary care. 

Maintaining a multidisciplinary team to treat these patients ensures that their care will involve the use of the most appropriate tools at the most appropriate times. Instituting just such a team of specialists from key disciplines will facilitate the incorporation of multiple points of view and treatment techniques that are most beneficial to patients with HCC. This multifaceted approach should provide the best possible outcomes for this patient population. This article is meant to review outcomes, options, and toxicity to serve as a resource for all those disciplines.

## Figures and Tables

**Table 1:** Recommendations from selected guidelines for the treatment of patients with unresectable HCC.

	AASLD/ESMO-EORTC [4,7]	NCCN [6]	HKLC [8]
**Definition of unresectable disease**	In patients with Child–Pugh A-B liver function and ECOG PS 0 - Single nodule in a patient with increased bilirubin - 3 nodules ≤3 cm Multinodular disease Portal vein invasion Extrahepatic spread ECOG PS 1–4	Inadequate liver function (i.e., Child–Pugh B or higher or Child–Pugh A with portal hypertension) Multiple masses Vascular invasion Inadequate future liver remnant after surgery	In patients with no extrahepatic vascular invasion/metastases - Intermediate tumor ^a^ with Child–Pugh B liver function, ECOG PS 0–1 - Locally advanced tumor ^b^ with Child–Pugh A-B liver function and ECOG PS 0–1 Patients with extrahepatic vascular invasion/metastases and Child–Pugh A-B liver function Patients with ECOG PS 2-4 and Child–Pugh C liver function
**Recommendations for LRT**	Ablation - Single nodule ≤2 cm, Child–Pugh A liver function, and ECOG PS 0 in patients who are not candidates for transplant - Single nodule or three nodules ≤3 cm, Child–Pugh A–B, ECOG PS 0, increased portal vein pressure, and associated disease TACE: multinodular disease, Child–Pugh A-B, ECOG PS 0 TARE: not eligible for TACE or sorafenib	Consider for patients who are not candidates for surgery or as part of a bridge strategy for other curative therapies Ablation - The tumor and a surrounding margin of tissue can be treated (i.e., tumors ≤5 cm) - Accessible location without damaging major vessels or bile ducts, the diaphragm, or other organs TACE - Arterial blood supply to tumor can be isolated - Bilirubin ≤3 mg/dL, no PVT, Child–Pugh A or B TARE - Arterial blood supply to tumor can be isolated - Bilirubin ≤2 mg/dL, no PVT, Child–Pugh A or B EBRT - All tumors irrespective of location in patients with unresectable disease - Consider as an alternative to ablation, TACE, or TARE - May be appropriate for symptom control and/or prevention of complications from metastatic disease	Ablation - Early tumor ^c^, no extrahepatic vascular invasion/metastases, ECOG Ps 0–1, Child–Pugh A-B liver function TACE - Intermediate tumor, no extrahepatic vascular invasion/metastases, Child–Pugh B liver function, ECOG PS 0–1 - Locally advanced tumor, no extrahepatic vascular invasion/metastases
**Recommendations for systemic treatment**	Patients with portal vein invasion, extrahepatic spread, Child–Pugh A-B, ECOG PS 1–2 Not a candidate for curative or locoregional treatments due to stage migration	Sorafenib - Child–Pugh A (category 1) or B (category 2A) liver function - Extensive disease; patient not suitable for transplant - Not suitable for resection due to comorbidity or presence of metastases Lenvatinib - Child–Pugh A liver function Regorafenib - Child–Pugh A liver function - Progression on sorafenib Cabozantinib - Child–Pugh A liver function - Progression on sorafenib Nivolumab - Child–Pugh A or B7 liver function - Progression on sorafenib Pembrolizumab - Child–Pugh A liver function - Progression on sorafenib Ramucirumab - AFP ≥ 400 ng/mL - Progression on sorafenib	Extrahepatic vascular invasion/metastases Child–Pugh A-B liver function, ECOG PS 0–1
**Recommendations for palliative care**	Child–Pugh C, ECOG PS 3–4 Extensive tumor Advanced liver disease	EBRT may be appropriate for symptom control and/or prevention of complications from metastatic disease Should be offered to patients with unresectable disease, metastatic disease, or extensive tumor burden	Extrahepatic vascular invasion/metastases ECOG PS 2–4, Child–Pugh C liver function

AASLD, American Association for the Study of Liver Disease; EBRT, external beam radiation; ECOG PS, Eastern Cooperative Oncology Group performance status; ESMO-EORTC, European Society for Medical Oncology-European Organization for Research and Treatment of Cancer; HKLC, Hong Kong Liver Cancer; LRT, locoregional treatment; NCCN, National Comprehensive Cancer Networks; PVT, portal vein thrombosis; TACE, transarterial chemoembolization; TARE, transarterial radioembolization. ^a^ Intermediate tumor = ≤5 cm, >3 nodules, and no intrahepatic venous invasion or >5 cm, ≤3 nodules with intrahepatic venous invasion. ^b^ Locally advanced tumor = ≤5 cm, >3 nodules with intrahepatic venous invasion, or >5 cm, >3 tumor nodules or/and with intrahepatic venous invasion, or diffuse tumor. ^c^ Early tumor = ≤5 cm, ≤3 tumor nodules and no intrahepatic venous invasion.

**Table 2:** Summary of recent studies on multidisciplinary care in unresectable HCC.

Study	Design	Key Findings
Charriere et al, 2017 [27]	All patients identified for treatment with transplant, resection, or RFA were prospectively enrolled (N = 387)	Compliance with the multidisciplinary team’s recommendations was associated with longer OS (HR 0.39 (95% CI 0.27–0.54), *p* < 0.0001) Factors associated with greater compliance with the multidisciplinary team’s recommendations included MELD score < 10, time frame of <60 between the decision and first treatment, platelet levels >126,000/mm^3^, and a decision for resection or RFA
Serper et al, 2017 [26]	Review of Veterans Administration clinical data repository (N = 3988)	Evaluation by >1 specialist was significantly associated with a higher likelihood of receiving active therapy (OR 1.60 ([95% CI 1.15–1.21)) Presence of a multidisciplinary team in the provider facility was associated with longer OS (HR 0.83 (95% CI 0.77–0.90))
Yopp et al, 2014 [25]	Patients diagnosed before and after the establishment of a multidisciplinary clinic were retrospectively evaluated (N = 355)	Patients in the multidisciplinary clinic were diagnosed at an earlier stage and presented with fewer symptoms and better ECOG PS than those treated before the clinic was established Potentially curative treatment was more likely to be given in the multidisciplinary clinic, with a significantly longer median OS than those treated before the clinic was established (13.2 months vs. 4.8 months (*p* = 0.005)
Chang et al, 2008 [24]	Survival of patients treated before and after the implementation of a multidisciplinary team was compared N=121 treated by the multidisciplinary team	Rate of patients referred to surgical services doubled after implementing the multidisciplinary tumor board, significantly increasing the number of patients treated in the early stages of the disease Survival rates were significantly higher in patients treated by the multidisciplinary team

ECOG PS, Eastern Cooperative Oncology Group performance status; HR, hazard ratio; MELD, Model for End-Stage Liver Disease; OR, odds ratio; OS, overall survival; RFA, radiofrequency ablation.
